# Telehealth virtual reality intervention reduces chronic pain in a randomized crossover study

**DOI:** 10.1038/s41746-025-01553-x

**Published:** 2025-04-07

**Authors:** Luana Colloca, Anna Han, Rachel Massalee, Nandini Raghuraman, Rachel L. Cundiff-O’Sullivan, Giancarlo Colloca, Yang Wang

**Affiliations:** 1 Department of Pain Translational Symptom Science, School of Nursing, University of Maryland, Baltimore, MD USA; 2 Center to Advance Chronic Pain Research, University of Maryland, Baltimore, MD USA; 3 Department of Anesthesiology and Psychiatry, University of Maryland School of Medicine, Baltimore, MD USA; 4 Placebo Beyond Opinions Center, University of Maryland School of Nursing, Baltimore, MD USA; 5 Department of Computer & Information Sciences, Information Technology PhD Program, Towson University, Towson, MD USA

**Keywords:** Psychology, Medical research, Signs and symptoms

## Abstract

The efficacy of telehealth Virtual Reality (VR) for chronic pain, a promising digital intervention, remains underexplored due to methodological challenges. In a 5-week crossover trial, we compared VR to matched audio content control in individuals with chronic pain. VR significantly reduced pain intensity, anxiety, and pain interference while improving mood and sleep quality. Findings highlight the potential of telehealth-based VR for addressing real-world challenges in managing chronic pain. ISRCTN12473220 (07/18/2023).

Chronic pain imposes a significant burden on both the individual and society, making it essential to consider the cost-effectiveness of digital therapeutics in comparison to the annual expenditures associated with chronic pain disorders. Pharmacological treatments make up $16.4 billion of the total annual cost of chronic pain treatment^1^, but they are often ineffective and cause diverse side effects, including opioid addiction^2^. Thus, there is a need for effective and accessible non-pharmacological treatments for chronic pain.

Immersive virtual reality (VR) therapy has gained attention as a non-pharmacological intervention, with evidence supporting its effectiveness in reducing chronic pain. In particular, VR has been successfully used to manage low back pain^3–9^, with AppliedVR’s EaseVRx becoming the first VR program cleared by the U.S. Food and Drug Administration (FDA) as an adjunct treatment for chronic lower back pain^10^.

Telehealth, in particular extended VR^11^, can deliver medical services remotely and improve healthcare access for individuals with mobility challenges or living in underserved areas. Indeed, a recent study^3^ evaluated a 56-day, self-administered, at-home, skills-based VR program for chronic low back pain (RelieVRx) in a large, demographically diverse, and clinically severe real-world sample. The study found significant long-term reductions in pain intensity and pain interference at 12 months post-treatment, demonstrating the potential of telehealth-based VR programs to deliver durable therapeutic benefits^3^.

Despite the promising application of VR, gaps remain in the literature. One issue is that previous clinical trials on the efficacy of VR for chronic pain^12,13^ used sham VR to control for immersion, but did not use the same content as the treatment condition, undermining the ability to isolate the therapeutic effects of VR-delivered mindfulness, breathing, and acceptance practices. Additionally, previous clinical trials lacked a non-intervention control condition, leading to difficulty in distinguishing the specific effects of VR from placebo responses^14^.

In this crossover study, we determined the effect of a telehealth-based VR intervention on chronic orofacial pain as compared to an audio-only (MP3) same-content control intervention^14^ and non-intervention. We also controlled expectations of pain relief. Interactions with participants were exclusively remote. All participants underwent a 5-day VR intervention and a 5-day MP3 intervention. The intervention order was counterbalanced. A 5-day non-intervention period followed each intervention (Fig. 1) to minimize carry-over effects.

**Fig. 1:**
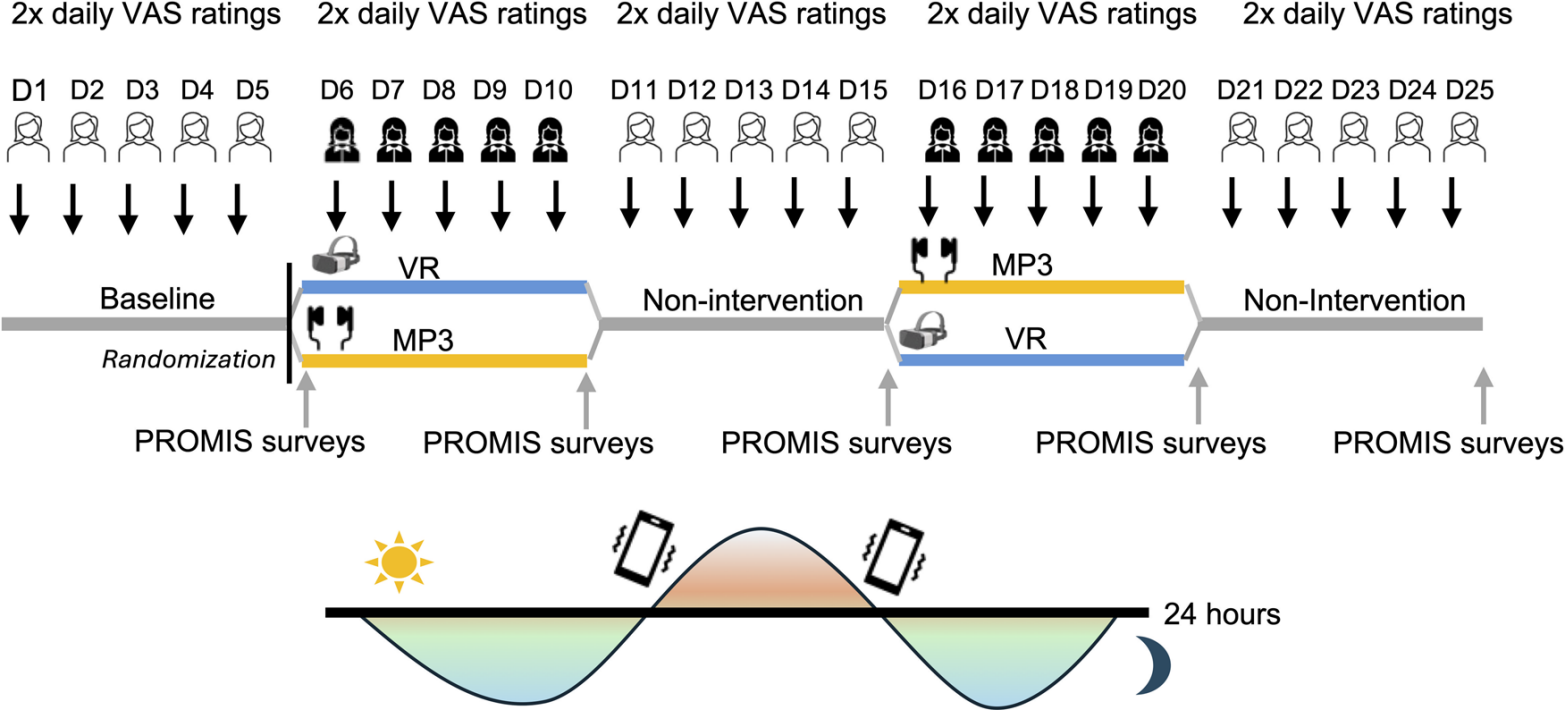
Randomized crossover trial design schematic. After the initial 5-day monitoring period, participants were randomized to complete either the MP3 intervention or the VR intervention first. Each intervention period was followed by a 5-day washout period to control for carry-over effects. Ecological momentary assessment was used for daily pain outcomes monitoring. Specifically, two survey prompts were sent to participants’ cell phones twice per day to measure the dynamics of pain fluctuations.

To capture day-to-day fluctuations of chronic pain outcomes, we applied an ecological momentary assessment (EMA) approach, using a HIPAA-compliant smartphone platform. We measured EMA-based pain outcomes using a visual analog scale (VAS) with a range from 0 to 100 twice daily, before and after the interventions. Similarly, we measured pain-related outcomes twice during the non-intervention. The remote evaluator was blind to randomization.

We hypothesized that the telehealth-based self-administered VR would result in greater pain reduction as compared to the MP3 same-content intervention in participants suffering from chronic orofacial pain.

Fifty-four patients with chronic orofacial pain who had a primary diagnosis of temporomandibular disorders (TMD), a common orofacial pain condition impacting about 30% of the general population^15^, participated in the study. We randomly assigned the participants (1:1 ratio) to either the VR intervention followed by MP3, or the MP3 intervention followed by VR. Fifty-three completed the trial (see Table 1). Baseline clinical pain was moderate, with an average Graded Chronic Pain Scale^16^ score of 2.04 out of 4.

**Table 1:** Sociodemographic and Clinical Characteristics of Study Participants

Characteristic	*N*/Mean ± SD	%/Range
**Age (years)**	40.9 ± 13.9	21–66
**Sex**		
Males	39	73.58
Females	14	26.42
**Race**		
Asian	3	5.66
African American	20	37.74
White	27	50.94
Other^a^	3	5.66
**Combined household income**		
$0–$19,999	10	18.87
$20,000–$39,999	6	11.32
$40,000–$59,999	14	26.42
$60,000–$79,999	4	7.55
$80,000–$99,999	7	13.21
$100,000–$149,999	8	15.09
$150,000 or higher	4	7.55
**Highest level of education**		
College graduate	23	43.4
Post-graduate	13	24.53
Some college	13	24.53
High school	4	7.55
**Marital status**		
Divorced	6	11.32
Living as married	1	1.89
Married	9	16.98
Never married	33	62.26
Separated	1	1.89
Widowed	3	5.66
**TMD type**^**b**^		
Myalgia	47	88.68
Myofascial pain with referral	0	0
Right arthralgia	37	69.81
Left arthralgia	36	67.92
TMD headache	26	49.06
**Pain/mental treatments**		
NSAID	14	26.42
Muscle relaxants	2	3.77
BDZ	3	5.66
TCA	0	0.00
SSRI	7	13.21
SNRI	3	5.66
SARI	2	3.77
NDRI	5	9.43
**Chronic overlapping pain conditions**		
Knee pain	6	11.32
Shoulder pain	6	11.32
Low back pain	12	22.64
Osteoarthritis	9	16.98
Fibromyalgia	1	1.89
Headache	35	66.04
Migraine	10	18.87
**Blood pressure (mm Hg)**		
Systolic	127.21 ± 14.47	98–158
Diastolic	82.28 ± 11.62	64–121
**Heart rate (bpm)**	74.02 ± 12.51	52–107
**BMI**	29.71 ± 7.80	17.0–49.7
**GCPS** (0–4)	2.04 ± 1.07	1–4
**JFLS-20** (0–20)	2.29 ± 2.16	0.00–7.67
**OBC** (0–85)	29.87 ± 14.02	0.00–74.00
**Pain duration** (months)	134.38 ± 123.94	3–480
**PROMIS** (Baseline)		
Pain interference	61.29 ± 5.43	53.8–78.3
Pain behavior	50.93 ± 7.13	34.1–62.2
Sleep	54.7 ± 9.33	28.9–76.5
Anxiety	59.02 ± 7.52	36.3–74.3
**Mood disorders**		
BDI score (0–63)	12.79 ± 9.65	0–40
STAI score (20–80)	43.79 ± 11.37	20–71

Abbreviations: *BMI* body mass index, *GCPS*grades of chronic pain scale, *JFLS* jaw function limitation scale, *OBC* oral behavior checklist, *NSAID* non-steroidal anti-inflammatory drugs, *BDZ* benzodiazepine, *TCA* tricyclic antidepressants, *SSRI* selective serotonin reuptake inhibitors, *SNRI* selective serotonin-norepinephrine reuptake inhibitor, *SARI* serotonin antagonist and reuptake inhibitors, *NDRI* norepinephrine–dopamine reuptake inhibitor, *BDI* Beck Depression Inventory, *STAI* state trait anxiety inventory.

^a^Other race included 2 mixed races and 1 Native American.

^b^TMD types often overlapped.

The primary outcome was the EMA-based pain intensity with daily post-minus-pre-intervention VAS ratings with a range of *0* *=* *no pain* to *100* *=* *maximum pain*. The secondary outcomes were EMA-based post-minus-pre-intervention VAS ratings for pain unpleasantness (0 = no pain unpleasantness to 100 = maximum pain unpleasantness), anxiety (0 = not anxious to 100 = very much anxious), and mood (0 = worst mood to 100 = best mood). Other explorative outcomes included Patient-Reported Outcomes Measurement Information System (PROMIS)-measured pain interference^17^, pain behavior^18^, anxiety^19^, and sleep disturbance^20^ at the end of each 5-day intervention (see Fig. 1). Both primary and secondary outcomes were normally distributed. We provide details regarding missing data handling and outcomes distributions in Supplementary Materials.

We conducted linear mixed model analyses with daily change (post-minus-pre-intervention) in pain intensity as a dependent variable assessed over 5 days, controlling for baseline (5 days) and sequence of intervention. There were no statistically significant differences for any of the measures for the two non-intervention periods. Thus, we combined the data for a single non-intervention condition (see Supplementary Materials).

We observed a significant main effect of experimental condition (non-intervention, MP3, VR) on changes in pain intensity (*F*_2,464.57_ = 14.29, *p* < 0.001, Cohen’s *d* = 0.48, 95% CI = 0.12 to 0.89, Fig. 2a). Bonferroni-corrected post-hoc comparisons indicated that VR induced greater pain reductions than both the MP3 comparator (*p* = 0.003) and non-intervention (*p* < 0.001). The number needed to treat (NNT) for the efficacy of VR in comparison with the MP3-based intervention was 3.76.

**Fig. 2:**
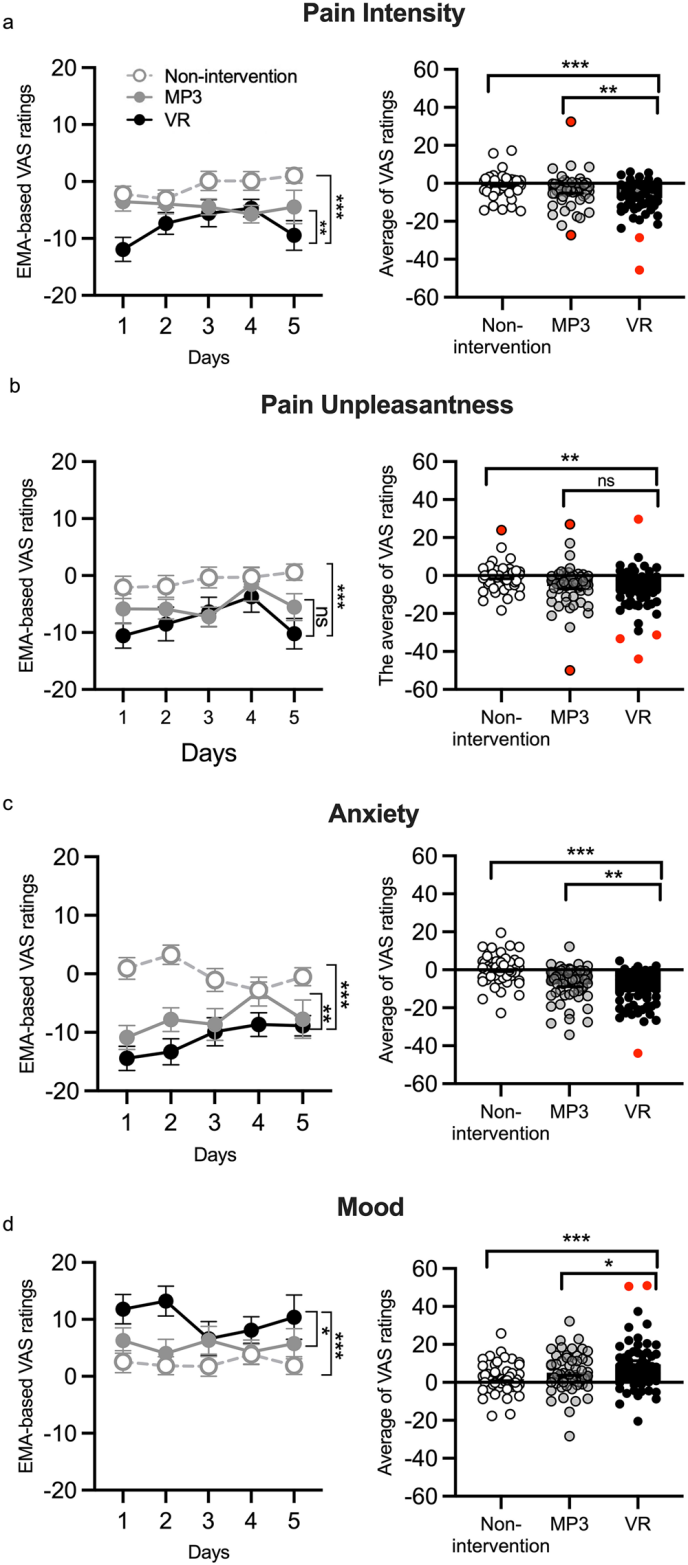
Delta scores for primary (pain intensity) and secondary (pain unpleasantness, mood, anxiety) outcomes. There were significant differences between VR and MP3 intervention delta scores for pain intensity (**a**), pain unpleasantness (**b**), situational anxiety (**c**), and mood (**d**). The mean and standard error of the mean (SEM) were plotted for each condition. Outliers identified by the Tukey formula were marked in red. *p < 0.05; **p < 0.01; ***p < 0.001.

For secondary outcomes, we found a main effect of experimental condition on pain unpleasantness (*F*_2,451.06_ = 12.66, *p* < 0.001, Fig. 2b), anxiety (*F*_2,446.44_ = 45.16, *p* < 0.001, Fig. 2c), and mood (*F*_2,451.94_ = 10.55, *p* < 0.001, Fig. 2d). For pain unpleasantness, post-hoc analyses showed that the VR condition was significantly different from non-intervention (*p* < 0.001) but not the MP3 condition (*p* = 0.127). For anxiety reduction and mood improvement, the VR condition was significantly different from MP3 (anxiety: *p* = 0.002; mood: *p* = 0.011) and non-intervention (anxiety: *p* < 0.001; mood: *p* < 0.001). Data are provided in Supplementary Table 1.

There were also significant differences between the experimental conditions for PROMIS-measured pain-related dysfunction at the end of each 5-day intervention or non-intervention. There were significant effects of experimental condition on pain interference (*F*_2,70.96_ = 4.87, *p* < 0.001, Fig. 3a), pain behavior (*F*_2,52.73_ = 3.62, *p* = 0.034, Fig. 3b), general anxiety (*F*_2,74.48_ = 3.77, *p* = 0.02 Fig. 3c), and sleep quality (*F*_2, 82.20_ = 5.26, *p* = 0.007, Fig. 3d). Post-hoc Benjamini–Hochberg corrected analyses showed that VR significantly decreased pain interference (MP3: *p* = 0.009, non-intervention: *p* = 0.050); pain behavior, which measures the extent of an individual’s external pain manifestations (MP3: *p* = 0.030, non-intervention: *p* = 0.041); general anxiety (MP3: *p* = 0.042, non-intervention: *p* = 0.033); and sleep quality (MP3: *p* = 0.006, and marginally lower than non-intervention: *p* = 0.075).

**Fig. 3:**
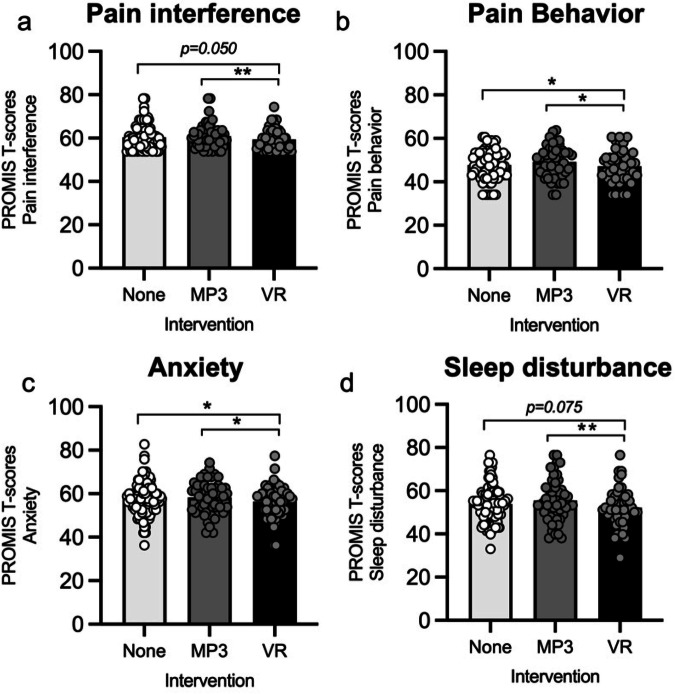
Secondary outcomes: weekly PROMIS T-scores. Significant differences between VR and MP3 intervention T-scores were observed for pain interference (**a**), pain behavior (**b**), anxiety (**c**), and sleep disturbance (**d**). The y-axes reflected the standardized PROMIS T-score ranges specific to each outcome, rather than a uniform 0–100 scale. The mean and standard error of the mean (SEM) were presented. *p < 0.05, **p < 0.01, 0 ***p < 0.001.

We also investigated factors influencing VR intervention response, focusing on demographic and clinical variables. Older age correlated with greater VR-induced pain reductions (Spearman *r* = −0.38, *p* = 0.006). Sex and race did not affect VR outcomes. Regarding clinical variables, we examined the impact of high- vs. low-impact pain, defined by GCPS grades, on VR effects. Participants with high-impact pain showed significantly greater pain reductions across all conditions (*F*_1,48.97_ = 8.71, *p* = 0.005). Chronic overlapping pain conditions showed no influence. However, VR was effective only in participants without depression (Beck Depression Inventory < 14, *F*_2,436.23_ = 3.47, *p* = 0.032) and without anxiety (State-Trait Anxiety Inventory < 38, *F*_2,453.92_ = 4.47, *p* = 0.012). Further details on the moderating effects of these variables are in the Supplementary Materials. Participants reported no adverse reactions or complications associated with the VR or MP3 interventions.

To facilitate retention, we conducted an educational session immediately after the informed consent process. We showed participants a 2-min presentation depicting the effects of VR from our previous studies (Supplementary Materials). Before and after, we assessed expectations of VR-induced pain relief on a 100 VAS scale. Education significantly increased expectations of pain relief as compared to baseline expectations (*F*_1,51_ = 23.47, *p* < 0.001, Fig. 4a). Independent of education, treatment expectations were not correlated with VR-induced pain intensity or unpleasantness changes (all *p* > 0.128, Fig. 4b). Baseline (Spearman *r* = −0.27, *p* = 0.048) and post-education (Spearman *r* = −0.30, *p* = 0.029) expectations of pain relief predicted greater VR-induced reductions for situational anxiety (Fig. 4c). Additionally, greater post- but not pre-education expectations predicted greater VR-induced improvements in mood (Spearman *r* = 0.28, *p* = 0.044, Fig. 4d).

**Fig. 4:**
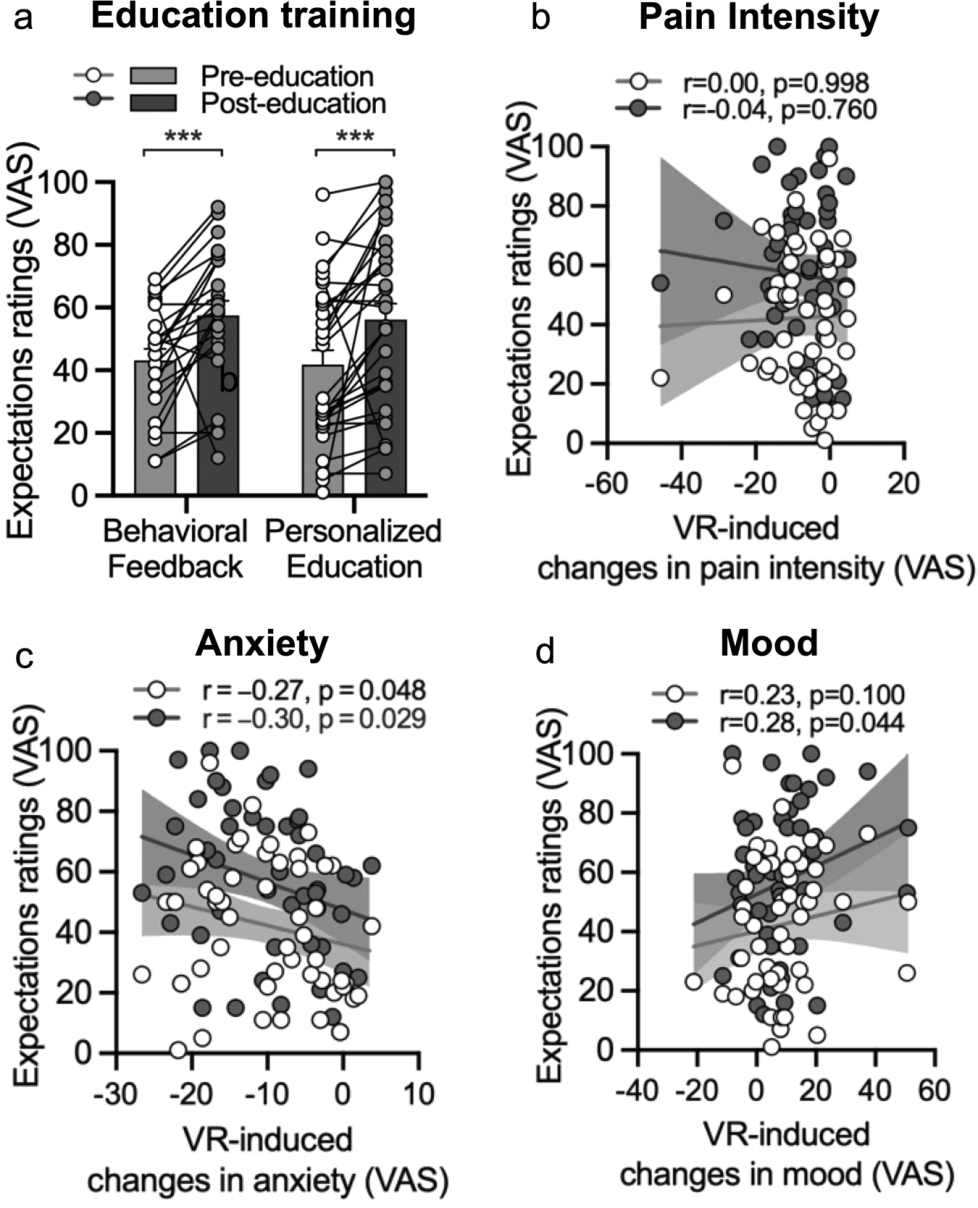
Effect of education training on expectations. **a** Educational interventions effectively enhanced participants’ expectations about VR’s benefits. Behavioral Feedback Approach: Participants (*n* = 23) had previously completed a one-session VR study and were shown their individual data, reflecting their VR-induced improvements. Personalized Education Approach: Participants (*n* = 30) without prior VR study experience were shown group-level results from the one-session VR study and a participant matched to the viewer’s age, sex, and race. Both education sessions improved participants’ expectations of pain relief. **b** Pre- and post-education expectations of pain relief were not associated with greater reductions in pain intensity. **c** Pre- and post-education expectations predicted situational anxiety improvements following VR exposure. **d** Stronger post-educational expectations, but not baseline expectations, were associated with improved mood outcomes.

This crossover trial demonstrated that a telehealth-based VR program not only improved clinical pain intensity, but also had significant effects on pain interference, pain behavior, mood, and sleep disturbance in individuals with chronic TMD. These findings add to the growing evidence supporting VR as an effective intervention for managing chronic pain conditions, such as low back pain^12,13^ and now TMD.

Our study addresses key methodological limitations identified in previous VR trials^12,13^. Unlike previous studies that used sham VR to control for immersion but failed to employ consistent content across conditions, our trial utilized the same therapeutic content—focused on mindfulness, breathing, and acceptance—in both active VR and MP3 programs. This design allowed us to isolate the specific therapeutic effects of VR—minimizing confounding variables, enhancing the interpretability of our results, and providing a framework to address a gap in the literature. Another strength of our trial is its telehealth delivery model, which eliminated the need for in-person visits, thereby improving accessibility for patients. It should be noted that social isolation during the SARS-CoV-2 pandemic may have increased participant adherence. Additionally, older age correlated with better pain reduction, aligning with findings of positive outcomes in older adults^21,22^. VR is a one-time investment with minimal upkeep, offering scalable, non-invasive pain relief and reducing long-term healthcare costs and medication reliance.

Our trial addressed the gap in using same-content interventions by focusing on short-term outcomes, as VR effects are often transient. However, studies have shown that VR interventions used continuously over 21 days or 56 days can yield lasting durability, with effects persisting for up to 18–24 months^4,5^. Despite the relatively short duration of our 5-week trial, our results highlight VR’s potential to deliver meaningful therapeutic benefits beyond placebo effects^14^. Future research should explore the cost-effectiveness, tech literacy, psychographics, and long-term safety profile of VR interventions to better assess their place in clinical practice.

## Methods

54 participants with chronic temporomandibular disorders (TMD) were enrolled (age = 40.9 ± 13.9; 39 women, 14 men; see Table 1). The study was conducted from November 23, 2020, to May 11, 2021, and was performed virtually without any in-person visits (see CONSORT Flow Chart in Supplementary Materials). The trial was registered in the International Standard Randomized Controlled Trial Number (ISRCTN) registry with the identifier ISRCTN12473220 on July 18, 2023. This clinical trial was approved by the University of Maryland, Baltimore Institutional Review Board (IRB Protocol #HP-00069094). All procedures were conducted in accordance with the Declaration of Helsinki and the International Conference on Harmonization Good Clinical Practice guidelines. All participants provided their written (e-consent) and verbal (via Zoom) consent to participate. All participants were made aware that their participation was voluntary and that they had the right to withdraw. Only one participant withdrew due to a lack of time to continue the trial. As compensation, participants received $220 in the form of either a check or an electronic gift card. Immediately after signing the informed consent, participants were introduced to the potential benefits of VR through a behavioral feedback or personalized education approach to encourage engagement and retention (see Fig. 4).

### Study design

This was a randomized crossover clinical trial design, with each participant completing both VR and MP3 control interventions in a fully counterbalanced and randomized order. To eliminate carry-over effects, a 5-day no-intervention period was added after each of the VR and MP3 control interventions. The trial took place over the course of 5 weeks. After signing the electronic consent form, all participants started the trial with a pre-intervention 5-day baseline period. During this time, participants were trained to use their cell phones to answer the daily surveys sent via Qualtrics twice per day (one in the morning and one in the afternoon).

We generated the randomization list using the MATLAB randomization function. Half of the participants completed the VR intervention followed by the MP3 intervention, while the other half completed the MP3 followed by VR.

Participants were randomly assigned to start with either the VR or the MP3 intervention after the baseline assessment. Neither the study team nor the participants knew the randomization until the completion of the pre-intervention 5-day baseline assessment. The sequence assignment was randomized according to a 1:1 ratio for the VR-first and the MP3-first groups.

This trial was conducted as an open-label study, in which both the study team and participants were aware of the assigned interventions (VR, MP3, and non-intervention). However, the remote evaluator, the principal investigator, and the statistician remained blind to the randomization.

### VR and MP3 interventions

EaseVRx (AppliedVR, Inc.) was used to deliver the VR intervention. EaseVRx is an immersive intervention comprising comprehensive multi-disciplinary modules for chronic pain management, including both psychological (cognition and emotion regulation, mindfulness, distraction) and physiological (diaphragmatic breathing, biofeedback, relaxation) methods. The VR content consisted of 10 relaxing experiences featuring ambient or soothing music. For example, participants experienced guided relaxation and interoceptive techniques (e.g., “Attracting abundance”, “Body relaxation”, “Letting go”, “Love and care”, “Breath awareness”). Some experiences did not include guidance but instead featured ambient naturalistic music, such as “Sun and clouds”, “Bavarian Alps”, “Tibet singing bowls”, “Healing with dolphins”, and “Dream beach.” These 10 modules were pre-selected from the device based on our prior findings with VR^23^, in which relaxing, immersive content with ambient music demonstrated the strongest effects in attenuating pain.

The MP3 content was identical to the audio of the VR intervention, except that MP3 did not contain any visual components and was delivered through an MP3 device. The participants were asked to put on earphones for the MP3 intervention. The comparison between the VR and MP3 interventions allowed us to quantify the effects of immersion and a sense of presence in improving chronic-pain-related outcomes.

The inclusion of both VR and MP3 interventions aimed to explore the differential effects of immersive (multi-sensory) versus non-immersive (auditory-only) relaxation techniques on chronic pain. This design also facilitated a comparison between the engagement levels and adherence rates of the two modalities.

#### Implementation

Participants were instructed to complete sessions daily over the intervention period, ensuring standardized exposure to the 10 pre-selected modules. For each intervention, participants were asked to complete 20 min worth of VR or MP3 sessions every day for a total of 5 days. We allowed them to rest during weekends.

A Zoom call between the experimenter and the participant was launched at the beginning of each study period, resulting in three Zoom calls: at enrollment, at the beginning of the VR intervention, and at the beginning of the MP3 intervention. Upon completion of the trial, participants were instructed to mail back the intervention devices using the return label provided to them. All postage costs were covered by the study team. Adherence and session completion were tracked through a session log diary (Supplementary Fig. 2).

### Population

We confirmed the TMD diagnosis of each participant at the University of Maryland Baltimore Brotman Facial Pain Clinic, School of Dentistry through an in-person clinical examination by an independently trained examiner, according to the Axis I Diagnostic Criteria (DC/TMD)^24^.

Eligibility for the study required being between 18 and 88 years old, understanding written and spoken English, and having had TMD for at least three months. Exclusion criteria included the following diseases: degenerative neuromuscular, cardiovascular, neurological, pulmonary, or kidney disease; cancer; personal or family first-degree history of severe psychiatric conditions; cervical spinal pain other than TMD; use of antidepressants, attention-deficit/hyperactivity disorder medication, or prescription painkillers; alcohol or drug dependence; pregnancy or breastfeeding; color-blindness; jaw or temple pain in the last three months due to toothache, infection, or facial trauma; impaired or uncorrected hearing; and contraindications for VR (e.g., history of severe motion sickness).

Sociodemographic variables, including age, sex, race, income, education, and marital status were assessed at enrollment. Chronic pain intensity and interference were measured using the graded chronic pain scale (GCPS)^16^, with greater values of GCPS indicating higher-impact pain. Given that chronic pain participants often experience concurrent mood disorders, we also assessed baseline levels of depression and anxiety using the Beck Depression Inventory (BDI)^25^ and the State-Trait Anxiety Inventory (STAI)-Trait^26^, respectively.

### Measurements

#### Ecological momentary assessment

We utilized an ecological momentary assessment (EMA) approach to assess daily and weekly changes in pain-related outcomes^27^ through the Qualtrics platform (see Supplementary Materials). Specifically, message prompts were sent to participants’ phones twice a day. Participants were instructed to complete the EMA questions when they received message prompts. A reminder was sent to the participant if the survey was not completed by 5 pm. The EMA allowed us to capture daily real-time fluctuations in clinical outcomes such as pain intensity, pain unpleasantness, mood, and anxiety by minimizing recall bias and maximizing ecological validity via random time sampling^27^. *Visual Analog Scales (VAS)*. We assessed expectations for pain relief before and after the expectation education sessions. Participants were asked to rate, “How much do you expect that VR will reduce your clinical pain?” on a 0–100 visual analog scale (VAS), with *0* = *No pain relief at all* to *100* *=* *Maximum pain relief*. This measurement provided both a baseline expectation rating and a reinforced expectation rating. Clinical pain intensity, pain unpleasantness, mood, and situational anxiety were measured at baseline and twice daily using VAS—pre- and post-intervention. During the baseline and no-intervention days, the first measurement was taken in the morning and the second measurement in the afternoon. The VAS ranged from 0 to 100, with *0* = *No pain/Not unpleasant/Worst mood/No anxiety* and *100 = Severe pain/Extremely unpleasant/Best mood/Severe anxiety*.

In addition to the daily VAS measurement, we assessed weekly patient-reported outcomes related to chronic pain using the Patient-Reported Outcomes Measurement Information System (PROMIS), a validated framework for measuring health domains across clinical populations. The specific PROMIS domains utilized in this study were: (1) *Pain interference*: Evaluated using the PROMIS Pain Interference item bank, which assesses the impact of pain on daily activities, social functioning, and emotional well-being. Scores reflect higher levels of pain interference^17^. (2) *Pain behavior*: Assessed through the PROMIS Pain Behavior item bank, which captures observable manifestations of pain such as grimacing, guarding, and verbal complaints. Higher scores indicate greater pain behavior^18^. (3) *Anxiety*: Measured using the PROMIS Anxiety short form, which evaluates symptoms such as worry, fear, and hyperarousal. Scores are standardized, with higher scores reflecting increased anxiety levels^28^. (4) *Sleep disturbances*: Captured using the PROMIS Sleep Disturbance short form, focusing on difficulty initiating or maintaining sleep and perceptions of sleep quality. Higher scores indicate more severe sleep disturbances^20^.

Each domain was operationalized via fixed short forms, allowing for precise, individualized measurement while minimizing participant burden. Scores were calculated as T-scores (mean = 50, SD = 10) based on general U.S. population norms, enabling standardized comparisons across domains.

### Outcomes

Daily changes in VAS pain intensity ratings were assessed as the primary outcome. Daily changes in VAS pain unpleasantness, mood, and situational anxiety were assessed as secondary outcomes. Weekly assessed PROMIS pain interference, anxiety, pain behavior, and sleep disturbance, were assessed as explorative outcomes.

A sample size calculation was conducted based on a large effect size for the main effect of the intervention (VR vs. MP3 vs. non-interventions), estimated based on our previous publication on VR effects in enhancing pain tolerance^23^. The sample size calculation indicated that 40 participants would be sufficient to achieve a statistical power of 0.8 at an alpha level of 0.05. Considering the longitudinal design of the current study and an anticipated 15% drop-out rate, the final sample size was adjusted to 54 participants. G*power was used for the sample size calculation for the study.

### Statistical methods

We tested the effects of the VR intervention against the MP3 comparator and non-intervention by running linear mixed models (LMM). The three conditions (VR vs. MP3 vs. non-intervention) were set as the fixed factor. In the LMMs, the interventions (VR vs. MP3 vs. non-intervention) and days were set as repeated measures. Random intercepts were applied to the model to account for intra-individual differences in the repeated measures^29^. The delta scores of the daily pain intensity, pain unpleasantness, mood, and situational anxiety measures were set as the outcomes for separate LMMs.

We further tested the effects of VR using the weekly PROMIS pain interference, anxiety, pain behavior, and sleep disturbance assessments. Similar LMMs were built with the three conditions set as the fixed factor. For all the above analyses, baseline ratings and intervention sequence (VR first vs. MP3 first) were set as covariates. In regard to the expectations ratings, we performed repeated-measure ANOVA with the two groups (behavioral feedback vs. personalized education) as the between-subjects factor, and the time-point (before vs. after the session) was set as the repeated measure. The intervention sequence was included as a covariate. We further used Spearman correlations to examine the relationship between VR benefit expectations and actual VR-induced improvements in pain-related outcomes (post-minus-pre-intervention VAS pain intensity, unpleasantness, situational anxiety, and mood). We applied the Bonferroni correction to the LMM analyses for the primary (VAS pain intensity) and secondary outcomes (e.g., pain unpleasantness, anxiety, mood, sleep). We used the Benjamini–Hochberg correction for the exploratory outcomes (e.g., PROMIS outcomes) to balance the risk of false positives with the risk of false negatives.

## Supplementary information

Supplementary Materials

## Data Availability

The data supporting the findings of this study are included in the supplementary materials. Individual data are available upon reasonable request to the corresponding authors.
