# Supplementary material for: Telehealth virtual reality intervention reduces chronic pain in a randomized crossover study
Source: NPJ Digit Med. 2025 Apr 7;8:192. doi: 10.1038/s41746-025-01553-x (PMC11976909; doi:10.1038/s41746-025-01553-x)
Supplement: Supplementary file 1 — Supplementary Materials [file 41746_2025_1553_MOESM1_ESM.pdf]

Supplementary materials for

Telehealth virtual reality intervention reduces chronic pain in a randomized crossover study

Luana Colloca[1,2,3,4], Anna Han[1], Rachel Massalee[1], Nandini Raghuraman[1], Rachel L. Cundiff-O'Sullivan[1], Giancarlo Colloca[5], Yang Wang[1,2,4]

1. Department of Pain Translational Symptom Science, School of Nursing, University of Maryland, Baltimore, USA
2. Center to Advance Chronic Pain Research, University of Maryland, Baltimore, USA
3. Department of Anesthesiology and Psychiatry, University of Maryland School of Medicine, Baltimore, USA
4. Placebo Beyond Opinions Center, University of Maryland School of Nursing, Baltimore
5. Information Technology PhD program, Towson University, Towson, USA

CONSORT FLOWCHART

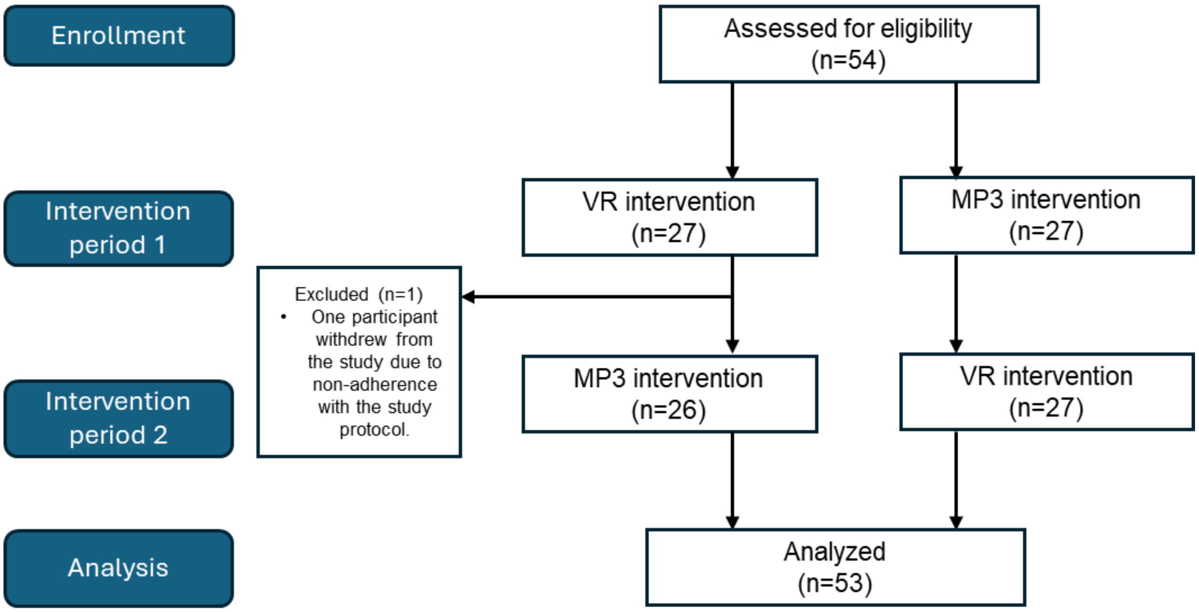

***Supplementary Figure 1. Tibet Singing Bowls**: A healing, meditative escape featuring soothing sounds of Tibetan singing bowls, designed to promote deep relaxation and mindfulness (left panel). **Dream Beach**: A peaceful, scenic escape on a serene beach, accompanied by gentle ambient sounds to evoke calmness and tranquility (right panel).*

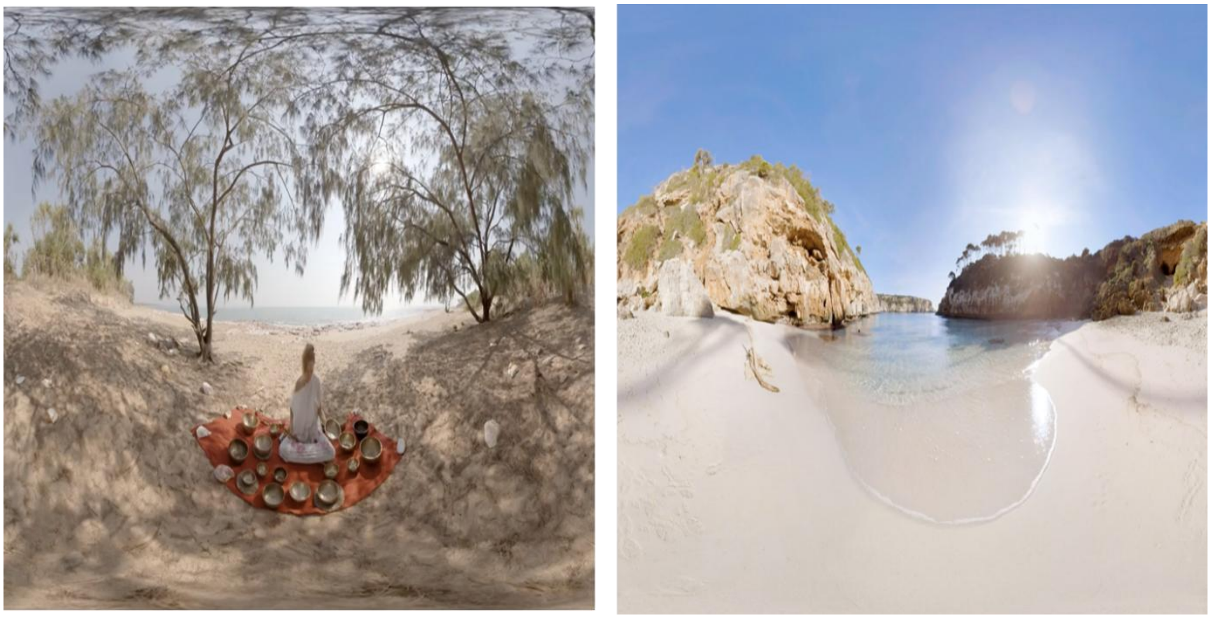

Examples of VR/MP3 contents:

Body relaxation:

"Let it melt into the horizon. Use the rhythm of your breath to help you explore where else you might be holding on. As you breathe in, if there are sensitive areas in your body, you may skip them or pay special attention to your breath as you scan for places of tension and fatigue. Let's start with your head. Notice your jaw.

Is it clenching? Let it drop. Let your tongue relax. Let your neck. Use your breath to wash away any tension.

Release and let go. Now allow your shoulders to release and drop. There is no burden for them to carry here. Let your breath pull them down so they can take a break. Now let your awareness travel down your arms and into your hands.

Let your fingers uncurl. Let your arms rest peacefully right where they are. There's nothing to hold on to. Release them now. Can you feel your back?

Bring your breath into your back, letting it wash over you now, coaxing the muscles to let go. Imagine your breath flowing between all the bones and muscles, giving them room to release. Let your body melt. There is no work to be done. Now bring your attention to your abdomen.

Take a breath and let the tension in your belly go with an H sound. In and H, H. Just let it out and make that little sound, five or six more times, H, H. Everything goes. And now focus on your legs and feet. They work so hard.

Take a breath in and exhale, let them relax. Let them, let it all. Now try to feel your whole body all at once. Are you more aware now of all its parts? Let your breath flow freely throughout your body, merging with each part.

Bringing relaxation wherever it goes."

Attracting abundance:

"Sometimes when we think of abundance, we think of financial abundance. However, we can also have an abundance of friendships, of health, of love and of happiness and learn how to attract abundance and live the life you have always dreamed of.

See yourself comfortably, breathe freely, easily, and effortlessly and let go of any worries or doubts, and as you breathe, think of the things you want in your life.

You repeat in your mind, I am Love and happy. I am successful and motivated. I am strong and healthy. I have friends who bring me joy and who I bring joy to.

We attract, what we focus on, and what you are feeling right now is the energy of abundance that You have attracted through the Power of your thoughts and as you continue to breathe, feel this energy, feel abundance tingling down your arms and into your fingers.

Feel it in your legs, in your feet and your toes. Feel it in every part of your body as it flows into the very depths of your soul.

Breathe deeply one last time, and as you breathe out, feel the happiness, the joy, the laughter and the fun carry over into every area of your daily life and every morning, when we wake up, we have a choice. You choose to have a good day every day."

Supplementary Figure 2. *The figure illustrates the materials provided to participants. Participants completed daily Ecological Momentary Assessments (EMA) via Qualtrics, using their personal smartphones, to log their adherence and subjective experiences (left panel of the Figure). Materials shipped to participants via FedEx included: VR goggles preloaded with EaseVRx modules, MP3 players with corresponding auditory modules, Instructions for device usage and disinfection, pre-paid shipping labels for returning devices, and Log diary to track session completion and reflections (right panel).*

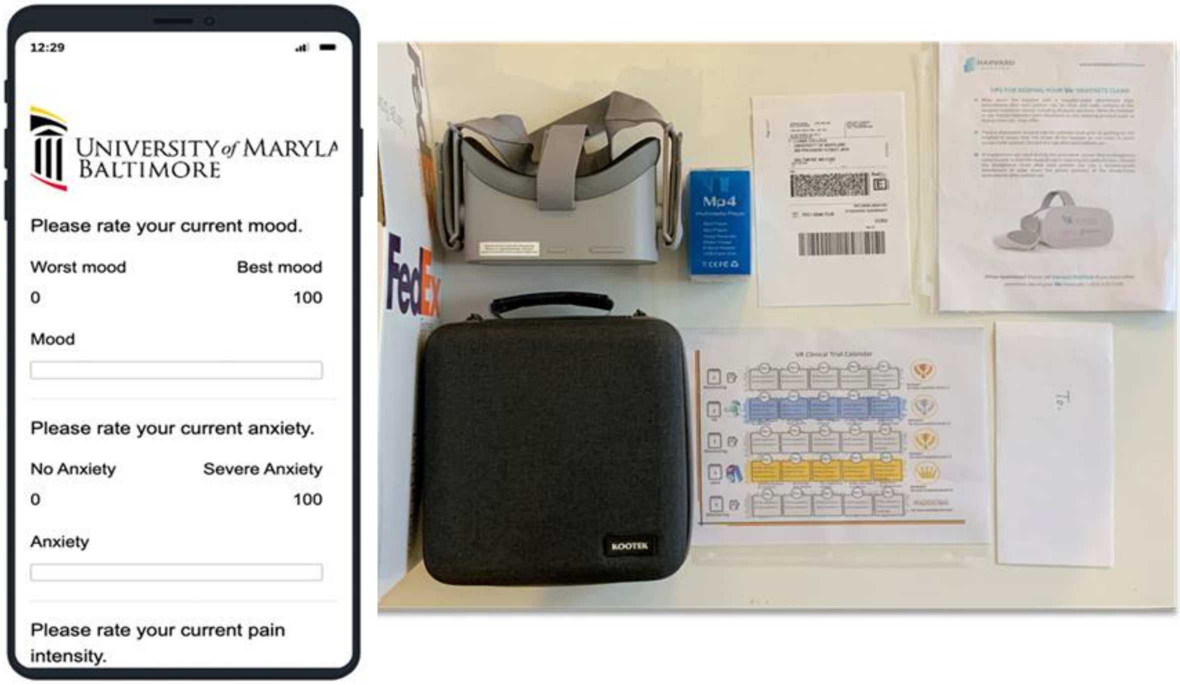

Supplementary Figure 3. *The session log diary. Participants completed a one-page diary to help track their participation in the trial and session completion. Upon completion of each intervention, rewards were also given (e.g. trophies).*

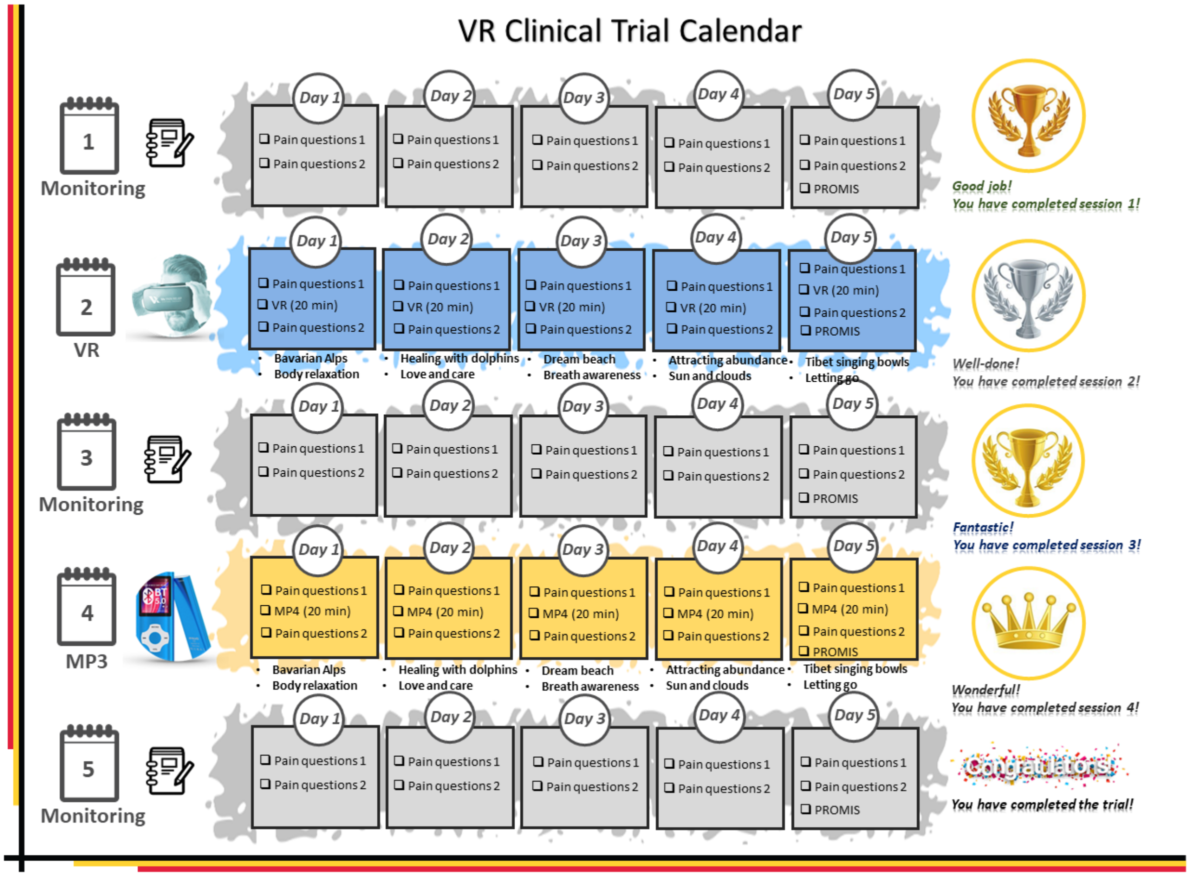

Supplementary Results

The EMA approach yielded a high compliance rate with 4.4% to 12.7% missing data, depending on different outcome measures. In addition, the missing data were randomly distributed with a non-significant Little's MCAR Test (chi-square=10.02, p=0.263). Linear mixed models were used given their superiority in addressing missing data structures.[1]

The data distribution and outliers were determined for the primary outcome (delta scores of daily VAS pain intensity ratings), secondary outcomes (i.e., delta scores of VAS pain unpleasantness, mood, situational anxiety), explorative outcomes (PROMIS pain interference, anxiety, pain behavior, and sleep disturbance), as well as changes in VAS expectations. Normal distributions were examined using histograms, kurtosis, and skewness scores. A kurtosis score between -7 and 7 and a skewness score between -2 and 2 were considered to be normal or close to normal distribution.[2] All the outcomes measured met the assumptions of normal distributions.

We identified outliers using the Tukey formula:

Lower limit = Q1 – 2.2X(Q3-Q1), Upper limit = Q3 + 2.2X(Q3-Q1).

When running the analyses excluding the outliers, all the results remained the same, except for VAS mood ratings, where the differences between VR and MP3 on mood changes were significant (p=0.038) but did not survive Bonferroni corrections (Bonferroni corrected p=0.111).

Intervention comparison for Phase I only. We conducted an additional analysis focusing exclusively on Phase 1 (VR vs. MP3) data, resulting in a between-subjects comparison. Specifically, the two groups (VR and MP3) were treated as a fixed factor, the 5-day period was treated as a repeated measure, and baseline measurements were included as covariates. Consistent with the main findings, after controlling for baseline variations, there was a significant main effect of group (Phase 1 VR vs. Phase 1 MP3) on changes in pain intensity ($F_{1,140.99}$ = 9.21, Bonferroni-corrected p = 0.003), pain unpleasantness ($F_{1,155.15}$ = 7.97, Bonferroni-corrected p = 0.005), and mood ($F_{1,168.68}$ = 4.17, Bonferroni-corrected p = 0.043), with a trend significance for anxiety ($F_{1,272.92}$ = 3.29, Bonferroni-corrected p = 0.071).

Moderating effects of sex, and race on VR impact on chronic pain. There was no significant main effect of sex ($F_{1,48.31}$=0.23, p=0.634) or the sex-by-condition interaction ($F_{2,446.30}$=0.20, p=0.818) on changes in daily pain intensity ratings, suggesting that sex did not impact VR effects on pain intensity reduction. Similarly, the main effects of race (White vs. non-White, $F_{1,48.81}$=0.76, p=0.389) and the race-by-condition interaction ($F_{2,453.81}$=1.41, p=0.245) were not significant, indicating that VR-induced pain attenuations were similar among participants of different races.

TMD is a common chronic overlapping pain condition. We further examined whether TMD in addition to other pain sites would have affected pain reductions induced by VR. Out of the 53 participants, 14 participants had TMD only, and the remaining 39 had TMD plus at least one chronic overlapping pain condition (e.g., headache, migraine, low back pain, knee pain, osteoarthritis, and fibromyalgia). There was no significant main effect of chronic overlapping pain conditions (TMD only vs. TMD + chronic overlapping pain conditions, $F_{1,48.15}$=0.53, p=0.471), nor its interaction with the condition ($F_{2,449.99}$=0.42, p=0.655). This result indicates that the presence of chronic overlapping pain conditions did not affect the VR-induced pain reductions.

Chronic pain is often comorbid with mood disorders such as depression and anxiety. The presence of depression was identified based on the Beck Depression Inventory (BDI) with the cut-off score of 14 out of 63.[3] 30 out of 53 participants had no depression, and 23 had mild to severe depression. There was a significant interaction between the presence of depression and condition ($F_{2,436.23}$=3.47, p=0.032). Posthoc analyses indicated that VR was only effective in reducing daily pain intensity within participants who had no depression (VR vs. MP3, -8.91 vs. -3.42, p=0.001; VR vs. Non-intervention, -8.91 vs. -1.99, p<0.001). Within the 23 participants who had mild to severe depression, VR did not differ from the MP3 (p=1.000) or the Non-intervention (p=0.151) in altering pain intensity.

We further identified presence of anxiety based on State-Trait Anxiety Inventory (STAI) scores with the cutoff score of 38.[4] There was a significant interaction between the presence of anxiety and condition ($F_{2,453.92}$=4.47, p=0.012). Posthoc analyses indicated that within

participants without anxiety, VR induced greater pain reductions (M=-11.27, SEM=2.10) than the MP3 (M=-2.14, SEM=1.83, p<0.001), and the Non-intervention (M=-2.40, SEM=1.75, p<0.001). Within participants who had moderate to high anxiety, VR did not differ from MP3 (p=1.000) in altering pain intensity, but both VR and MP3 conditions exhibited greater pain reductions than the Non-intervention condition (VR vs. Non-intervention: p=0.002; MP3 vs. Non-intervention: p=0.009).

No significant carry-over effects of VR and MP3. The two no intervention periods (no-VR: non-intervention after VR, and no-MP3: non-intervention after MP3) allowed us to examine the potential carry-over effects of VR and MP3 in altering pain-related outcomes. Specifically, linear mixed models were performed, treating the four time periods (VR vs. MP3 vs. no-VR vs. no-MP3) as the fixed factor. Changes in VAS pain intensity, pain unpleasantness, anxiety, and mood were set as dependent variables in each of the LMM analyses. The 5-day baseline assessments of pain intensity, pain unpleasantness, anxiety, and mood were treated as covariates, separately. Intervention sequence (VR-first vs. MP3-first) was also added as a covariate. All p-values were Bonferroni-corrected for multiple comparisons.

There were no carry-over effects of VR or MP3 for all the VAS measurements (**Supplementary Figure 4a**). Specifically, VR induced greater reductions in pain intensity ($F_{3,321.47}$=10.30, p<0.001) when compared to the MP3 (p=0.006), no-VR (p<0.001), and no-MP3 conditions (p<0.001). No differences were found between the no-VR and no-MP3 conditions (p=1.000).

In terms of pain unpleasantness, VR induced significantly larger reductions in pain unpleasantness than the two non-intervention periods ($F_{3,315.99}$=9.05, p<0.001, VR vs. no-VR: p<0.001; VR vs. no-MP3: p<0.001). However, VR did not differ from MP3 in terms of improvements in pain unpleasantness (p=0.255). No differences were found between the no-VR and no-MP3 conditions (p=1.000, **Supplementary Figure 4b**).

There was a significant main effect of the four conditions in altering mood ($F_{3,328.54}$=7.34, p<0.001). VR induced greater enhancements in mood as compared to MP3 (p=0.023) and the two non-intervention periods (p<0.001 for both). No differences were found between the no-VR and no-MP3 conditions (p=1.000, **Supplementary Figure 4c**).

Similar patterns were observed for daily anxiety ($F_{3,320.03}$=29.89, p<0.001): VR resulted in greater reductions in anxiety than MP3 (p=0.004) and the two no intervention periods (p<0.001 for both). No differences were found between the no-VR and no-MP3 conditions (p=1.000, **Supplementary Figure 4d**). Given the similarity in response during the two no intervention periods, we combined them into a single no intervention condition in the main analysis.

Supplementary Figure 4. *There were no carry-over effects of VR and MP3 in reducing pain intensity, enhancing mood, and improving anxiety. We observed no differences between the no intervention periods following VR and MP3 in changes in pain intensity (a), changes in pain unpleasantness (b), changes in mood (c), and changes in anxiety (d).*

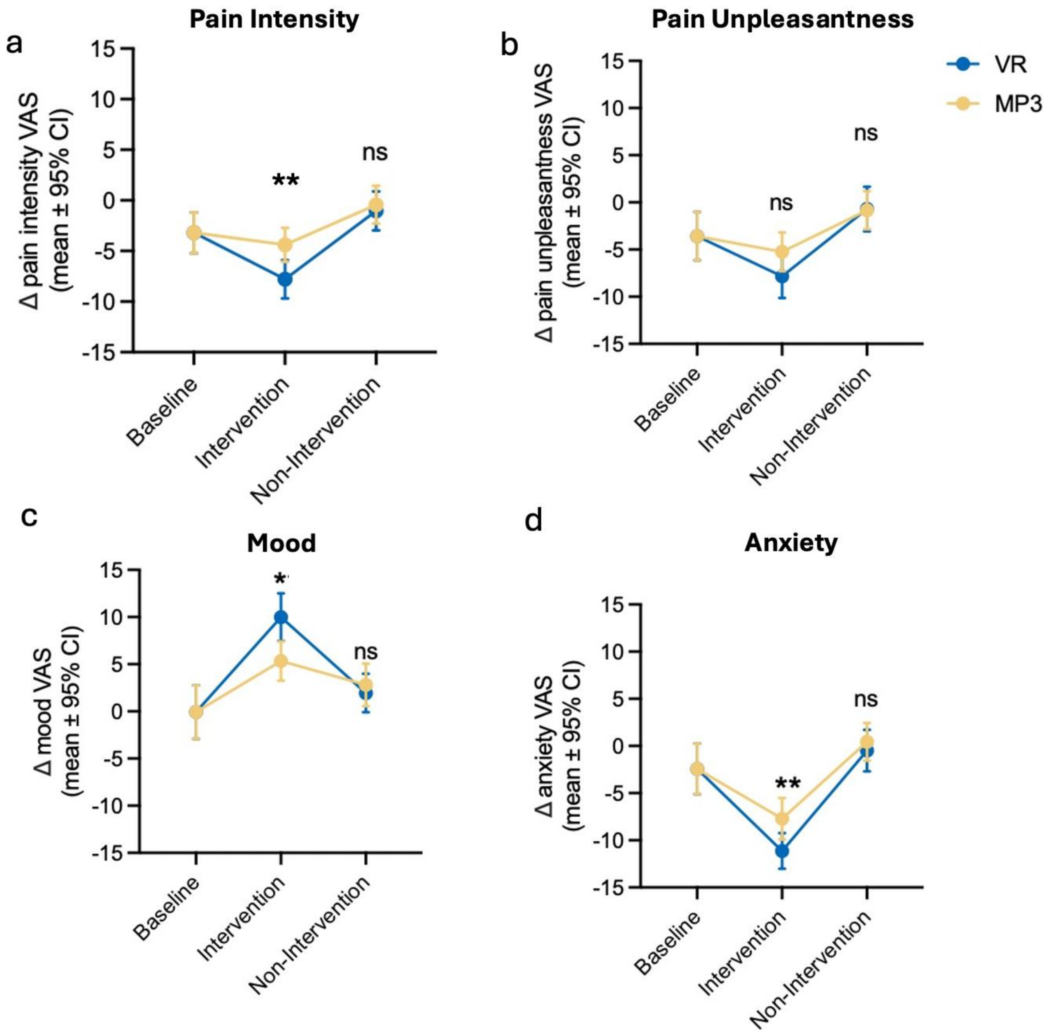

Supplementary Table 1. The average and standard error of mean of each experimental condition for the primary, secondary and explorative outcomes.

	Baseline		VR		Non-intervention following VR		MP3		Non-intervention following MP3	
	mean	sem	mean	sem	mean	sem	mean	sem	mean	sem
**VAS Pain Intensity**	-3.0667	0.98698	-7.7594	1.28376	-1.0495	0.9767	-3.9075	1.20429	-0.4346	0.9451
**VAS Pain Unpleasantness**	-3.0899	1.48225	-8.0651	1.60382	-0.6982	1.1994	-5.5522	1.49111	-0.8271	1.02064
**VAS Mood**	-0.666	1.32627	9.4704	1.89399	1.9414	1.03389	5.1544	1.43059	2.8084	1.13822
**VAS Anxiety**	-2.1164	1.35866	-11.7082	1.34039	-0.491	1.11668	-7.6535	1.24865	0.4299	1.01345
**PROMIS Pain Interference**	61.202	0.79604	59.3784	0.64787	60.3212	0.88316	60.9434	0.74621	60.3698	0.67906
**PROMIS Pain Behavior**	45.6755	2.22857	41.8113	2.27978	43.6736	1.97912	46.3906	1.83607	46.517	1.54054
**PROMIS Anxiety**	58.3904	1.15627	56.4078	1.04701	58.0115	1.08834	58.2434	0.99976	57.4906	1.02519
**PROMIS Sleep Disturbance**	54.8235	1.10679	52.2176	1.24127	54.1885	1.15031	55.5189	1.32615	53.4943	1.12681

Footnote: sem = standard error of mean

References

1. Chakraborty, H. & Gu, H. A mixed model approach for intent-to-treat analysis in longitudinal clinical trials with missing values. (2009).
2. Hair, J.F. Multivariate data analysis. (2009).
3. Gaynes, B.N., *et al*. Definition of treatment-resistant depression in the Medicare population. (2018).
4. Julian, L.J. Measures of anxiety. *Arthritis care & research* **63**(2011).

CONSORT 2010 checklist of information to include when reporting a randomised crossover trial*

Section/Topic	Item No	Checklist item	Reported on page No
**Title and abstract**			
	1a	Identification as a randomised crossover trial in the title	1
	1b	Structured summary of trial design, methods, results, and conclusions (for specific guidance see CONSORT for abstracts)	2
**Introduction**			
Background and objectives	2a	Scientific background and explanation of rationale	3
	2b	Specific objectives or hypotheses	3-4
**Methods**			
Trial design	3a	Rationale for a crossover design. Description of the design features including allocation ratio, especially the number and duration of periods, duration of washout period, and consideration of carry over effect	3-4, 8-10
	3b	Important changes to methods after trial commencement (such as eligibility criteria), with reasons	N/A
Participants	4a	Eligibility criteria for participants	10
	4b	Settings and locations where the data were collected	7-8
Interventions	5	The interventions for each group with sufficient details to allow replication, including how and when they were actually administered	9
Outcomes	6a	Completely defined pre-specified primary and secondary outcome measures, including how and when they were assessed	12
	6b	Any changes to trial outcomes after the trial commenced, with reasons	N/A
Sample size	7a	How sample size was determined, accounting for within participant variability	12
	7b	When applicable, explanation of any interim analyses and stopping guidelines	N/A
Randomisation:			
Sequence generation	8a	Method used to generate the random allocation sequence	4,8
	8b	Type of randomisation; details of any restriction (such as blocking and block size)	8
Allocation concealment mechanism	9	Mechanism used to implement the random allocation sequence (such as sequentially numbered containers), describing any steps taken to conceal the sequence until interventions were assigned	8
Implementation	10	Who generated the random allocation sequence, who enrolled participants, and who assigned participants to interventions	8

Blinding	11a	If done, who was blinded after assignment to interventions (for example, participants, care providers, those assessing outcomes) and how	4, 8
	11b	If relevant, description of the similarity of interventions	N/A
Statistical methods	12a	Statistical methods used to compare groups for primary and secondary outcomes which are appropriate for crossover design (that is, based on within participant comparison)	12-13
	12b	Methods for additional analyses, such as subgroup analyses and adjusted analyses	12
**Results**			
Participant flow (a diagram is strongly recommended)	13a	The numbers of participants who were randomly assigned, received intended treatment, and were analysed for the primary outcome	4,8
	13b	N of participants excluded at each stage, with reasons, separately for each sequence and period	Consort flow chart
Recruitment	14a	Dates defining the periods of recruitment and follow-up	4
	14b	Why the trial ended or was stopped	N/A
Baseline data	15	A table showing baseline demographic and clinical characteristics by sequence and period	Table 1
Numbers analysed	16	For each group, number of participants (denominator) included in each analysis and whether the analysis was by original assigned groups	4, Consort flow chart
Outcomes and estimation	17a	For each primary and secondary outcome, results for each group, and the estimated effect size and its precision (such as 95% confidence interval) should be based on within participants comparisons. In addition, results for each intervention in each period are recommended	5-6
	17b	For binary outcomes, presentation of both absolute and relative effect sizes is recommended	N/A
Ancillary analyses	18	Results of any other analyses performed, including subgroup analyses and adjusted analyses, distinguishing pre-specified from exploratory	Suppl. Materials
Harms	19	All important harms or unintended effects in each group (for specific guidance see CONSORT for harms)	6
**Discussion**			
Limitations	20	Trial limitations, addressing sources of potential bias, imprecision, and, if relevant, multiplicity of analyses. Consider potential carry over effects	6-7
Generalisability	21	Generalisability (external validity, applicability) of the trial findings	7
Interpretation	22	Interpretation consistent with results, balancing benefits and harms, and considering other relevant evidence	6-7
**Other information**			
Registration	23	Registration number and name of trial registry	1
Protocol	24	Where the full trial protocol can be accessed, if available	7
Funding	25	Sources of funding and other support (such as supply of drugs), role of funders	14

Citation: Schulz KF, Altman DG, Moher D, for the CONSORT Group. CONSORT 2010 Statement: updated guidelines for reporting parallel group randomised trials. BMC Medicine. 2010;8:18.
© 2010 Schulz et al. This is an Open Access article distributed under the terms of the Creative Commons Attribution License (http://creativecommons.org/licenses/by/2.0), which permits unrestricted use, distribution, and reproduction in any medium, provided the original work is properly cited.

*We strongly recommend reading this statement in conjunction with the CONSORT 2010 Explanation and Elaboration for important clarifications on all the items. If relevant, we also recommend reading CONSORT extensions for cluster randomised trials, non-inferiority and equivalence trials, non-pharmacological treatments, herbal interventions, and pragmatic trials. Additional extensions are forthcoming: for those and for up-to-date references relevant to this checklist, see www.consort-statement.org.
